# A Nucleolar Isoform of the *Drosophila* Ubiquitin Specific Protease dUSP36 Regulates MYC-Dependent Cell Growth

**DOI:** 10.3389/fcell.2020.00506

**Published:** 2020-06-19

**Authors:** Dominique Thevenon, Ilham Seffouh, Catherine Pillet, Xenia Crespo-Yanez, Marie-Odile Fauvarque, Emmanuel Taillebourg

**Affiliations:** Université Grenoble Alpes, CEA, INSERM, BGE U1038, Grenoble, France

**Keywords:** deubiquitinase (DUB), ubiquitin (Ub), MYC stability, CRISPR/Cas9, cell growth

## Abstract

The c-Myc oncogene is a transcription factor that regulates the expression of a very large set of genes mainly involved in cell growth and proliferation. It is overexpressed in more than 70% of human cancers, illustrating the importance of keeping its levels and activity under control. The ubiquitin proteasome system is a major regulator of MYC levels in humans as well as in model organisms such as *Drosophila melanogaster*. Although the E3 ligases that promote MYC ubiquitination have been largely investigated, the identity and the role of the deubiquitinating enzymes, which counteract their action is only beginning to be unraveled. Using isoform-specific CRISPR-Cas9 mutagenesis, we show that the *Drosophila* homolog of the Ubiquitin Specific Protease USP36 has different isoforms with specific sub-cellular localizations and that the nucleolar dUSP36-D isoform is specifically required for cell and organismal growth. We also demonstrate that this isoform interacts with dMYC and the E3 ligase AGO and regulates their stability and ubiquitination levels. Furthermore, we show that dUSP36 is ubiquitinated by AGO and is able to self-deubiquitinate. Finally, we provide *in vivo* evidence supporting the functional relevance of these regulatory relationships. Together these results reveal that dMYC, AGO and dUSP36 form a tripartite, evolutionary conserved complex that acts as a regulatory node to control dMYC protein levels.

## Introduction

The c-Myc oncogene encodes a pleiotropic transcription factor controlling the expression of a very large number of genes involved in differentiation, apoptosis, angiogenesis, metabolism, ribosomal biogenesis, cell growth and proliferation (van Riggelen et al., 2010; Sabo and Amati, 2014; Sabo et al., 2014). The expression level, stability and activity of MYC are tightly controlled to ensure proper cell growth and proliferation. c-Myc is overexpressed in the majority of human cancers and contributes to the cause of at least 40% of tumors (Nesbit et al., 1999; Dang et al., 2009; Lin et al., 2012; Miller et al., 2012). In mice, *Myc* overexpression drives tumorigenesis in a variety of tissues and *Myc* loss-of-function mutants are smaller, retarded in development, and fail to survive past embryonic day 9.5 (Davis et al., 1993). In *Drosophila*, partial loss-of-function mutations of the *Myc* ortholog (*dMyc* synonymous *diminutive*) result in delayed development and smaller than normal adult flies while null mutations strongly affect cell and organismal growth resulting in developmental lethality (Johnston et al., 1999; Pierce et al., 2004; Gallant, 2013; Grifoni and Bellosta, 2015).

In non-pathological conditions, MYC has a short half-life and is degraded by the ubiquitin-proteasome system (Farrell and Sears, 2014). The SCF^Fbw7^ complex, a SKP1-CUL1-F-box E3 Ubiquitin ligase complex where the Fbw7 F-box protein functions as the substrate recognition component, promotes MYC ubiquitination and degradation. Upon cell growth stimulation, MYC-SCF^Fbw7^ interaction is blocked by phosphorylation-dependent mechanisms leading to MYC stabilization and activation of cell growth and proliferation (Yada et al., 2004). Accordingly, *Fbw7* mutations are associated with multiple human cancers (Wang et al., 2014; Tong et al., 2017). The *Drosophila Fbw7* ortholog *Archipelago* (*Ago*) is also an important regulator of dMYC stability: loss-of-function mutations of *Ago* result in strongly elevated dMYC protein levels and increased tissue growth (Moberg et al., 2004).

Ubiquitination is a reversible modification: ubiquitin proteases, also known as deubiquitinases or deubiquitinating enzymes (DUBs), remove ubiquitin moieties from ubiquitinated proteins. In human cells, MYC is deubiquitinated and stabilized by two DUBs of the Ubiquitin Specific Protease (USP) family: USP28 (Amati and Sanchez-Arevalo Lobo, 2007; Popov et al., 2007) and USP36 (Sun et al., 2015a). These enzymes have specific roles regarding MYC since USP28 regulates MYC in the nucleoplasm (Popov et al., 2007) while USP36 regulates MYC in the nucleolus (Sun et al., 2015a). USP28 and USP36 each interact with specific isoforms of the E3 ligase sub-unit Fbw7. In *Drosophila*, the only DUB known to regulate dMYC stability is encoded by the *puffyeye* (*puf*) gene and is orthologous to human USP34 (Li et al., 2013). PUF interacts with the E3 ligase AGO and both proteins act antagonistically to regulate *dMyc* function in the developing eye and wing. While no obvious homolog of human USP28 is present in the *Drosophila* genome, USP36 has a clear *Drosophila* ortholog encoded by the *dUsp36* gene (Thevenon et al., 2009), also known as *scrawny* (*scny*) (Buszczak et al., 2009) or *emperor’s thumb* (*et*) (Ribaya et al., 2009). Its known functions include immunity (Thevenon et al., 2009; Taillebourg et al., 2014), stem cell maintenance (Buszczak et al., 2009), apoptosis (Ribaya et al., 2009), autophagy and cell growth (Taillebourg et al., 2012). dUSP36 has been shown to deubiquitinate histone H2B, which accounts for its role in stem cell maintenance (Buszczak et al., 2009), and the NF-kB pathway signaling protein IMD, which accounts for its role in immune signaling (Thevenon et al., 2009). However, the molecular causes of the cell and organismal growth defects observed in null *dUsp36* mutants (Taillebourg et al., 2012) remain to be characterized.

The aim of this study was to understand the role of dUSP36 in the regulation of cell and organismal growth and to identify the substrate(s) involved in this process. We first showed that the *dUsp36* gene produces three isoforms with different subcellular localizations when expressed in S2 cells: the dUSP36-C and -D isoforms are nuclear whereas the dUSP36-B isoform is cytoplasmic due to the presence of a specific nuclear export sequence. We then generated isoform-specific loss-of-function alleles by CRISPR-Cas9 mutagenesis (Jinek et al., 2012; Sternberg et al., 2014) and observed that the endogenous dUSP36-D isoform is localized in the nucleolus, as its human counterpart (Sun et al., 2015a), and plays a major role in cell and organismal growth with phenotypes similar to *dMyc* hypomorphic mutations. We then showed that the dUSP36-D isoform forms a complex with dMYC and AGO, regulating the stability and ubiquitination levels of both proteins. Furthermore, we observed that dUSP36-D is ubiquitinated by AGO and is able to self-deubiquitinate. These results indicate that dMYC, AGO and dUSP36 are part of the same macromolecular complex in which AGO ubiquitinates dUSP36 and dMYC while dUSP36 deubiquitinates itself, AGO and dMYC. We then provided genetic evidence supporting the functional relevance of these interactions during *Drosophila* development.

MYC regulation by the deubiquitinating enzyme USP36 as well as by the E3 ligase SCF^Fbw7^ have been described in human cells (Sun et al., 2015a) but were so far envisaged as acting independently. Our results show that, in *Drosophila*, dMYC is part of a tripartite complex containing both the E3 ligase AGO and the DUB dUSP36, which tightly controls its ubiquitination levels and stability. Given the conservation of the MYC regulatory network, it is likely that this complex also exists in human cells, which opens new avenues in understanding the regulation of MYC stability in physiological *versus* oncogenic conditions.

## Results

### The *dUsp36* Gene Encodes Three Isoforms With Different Subcellular Localizations

**FIGURE 1:**
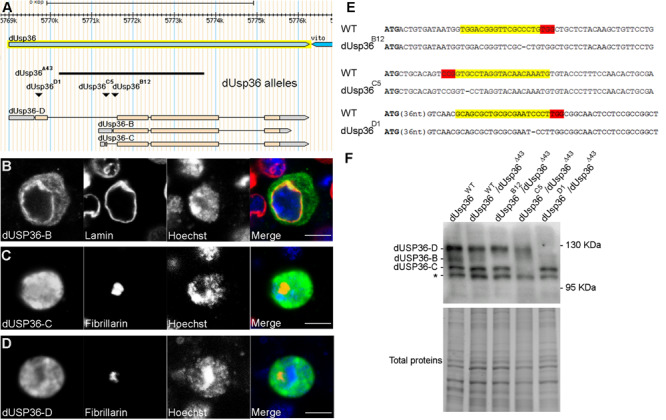
Subcellular localization of the dUSP36 isoforms and CRISPR-Cas9 mutagenesis. **(A)** Schematic representation of the *dUsp36* gene adapted from the Flybase *Drosophila* genome database. The genomic extent of the *dUsp36* gene is represented by the light blue arrow whereas the cDNAs corresponding to the different isoforms are represented by the gray (non-coding sequences) and pink (coding sequences) rectangles. The black bar shows the extent of the *dUsp36^Δ43^* deletion, which removes most of the *dUsp36* coding sequences and affects all three isoforms. The inverted triangles indicate the position of the one nucleotide deletion induced by CRISPR-Cas9 mutagenesis. **(B–D)** *Drosophila* S2 cells transfected with the indicated V5-tagged isoform and stained with anti-V5, anti-Lamin **(B)** or anti-Fibrillarin **(C,D)** antibodies and Hoechst are shown as separate gray channels and as merged images (Blue: Hoechst. Green: anti-V5 antibody. Red: anti-Lamin **(B)** or anti-Fibrillarin **(C,D)** antibodies). Scale bar: 5 μm. **(E)** The wild-type (WT) and mutant sequences corresponding to the dUSP36-B, -C and -D isoforms are respectively shown. The specific ATG used by each isoform is highlighted in bold. The gRNA sequences used for mutagenesis are highlighted in yellow whereas the PAM sequences are highlighted in red. Among the identified mutations (see Supplementary Figure S1 for an exhaustive list), a one nucleotide deletion that induces a frameshift mutation was selected for each isoform. **(F)** Total protein extracts from *Drosophila* males of the indicated genotype were analyzed by Western blot using a specific anti-dUSP36 antibody (Buszczak et al., 2009). In wild-type and heterozygous individuals, the three isoforms are expressed. The asterisk marks a non-specific band. The band corresponding to each isoform is not detected in the corresponding mutants whereas the other two are still expressed.

**FIGURE 2:**
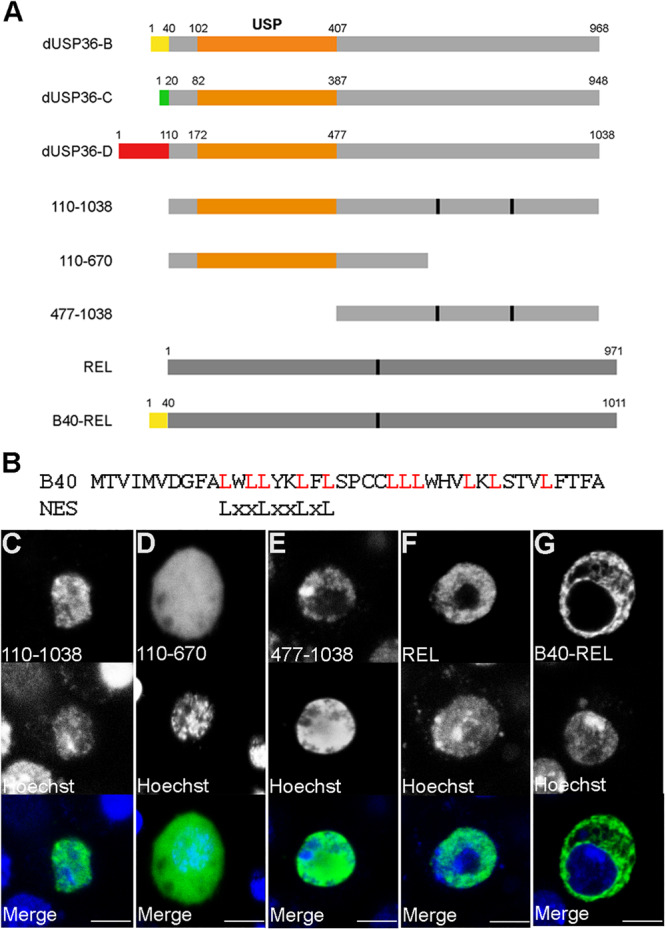
The specific N-terminal domain of the dUSP36-B isoform contains a Nuclear Export Sequence. **(A)** Schematic representation of the dUSP36 isoforms and of the constructs used in this study. The specific N-terminal domains are represented by colored rectangles. The common orange rectangle represents the USP catalytic domain. **(B)** Comparison of the sequence of the dUSP36-B specific N-terminal domain to the consensus sequence of CRM1-dependent NESs. **(C–G)** *Drosophila* S2 cells transfected with the indicated V5-tagged construct and stained with an anti-V5 antibody and Hoechst are shown as separate gray channels and as merged images (Blue: Hoechst. Green: anti-V5 antibody). Scale bar: 5 μm.

According to the Flybase *Drosophila* genome database (Gramates et al., 2017), the *dUsp36* gene encodes multiple putative transcripts but identification of full-length cDNAs supports the existence of only three of them (Figure 1A). The proteins expressed from these transcripts are identical except for their specific N-terminal domain (Figure 2A). The common part contains the Ubiquitin Specific Protease (USP) catalytic domain followed by a disordered domain (Gramates et al., 2017). When transfected into *Drosophila* S2 cells, V5-tagged isoforms display different subcellular localizations (Figures 1B–D): while the dUSP36-B isoform accumulates in the cytoplasm and at the nuclear membrane as shown by colocalization with Lamin (Figure 1B), the dUSP36-C and -D isoforms are localized in the nucleus (Figures 1C,D). However, under these overexpression conditions and in contrast to human USP36 (Endo et al., 2009; Sun et al., 2015a), their localization is not restricted to the nucleolus, highlighted by the nucleolar marker Fibrillarin, but expands to the whole nucleoplasm. To gain insight into the mechanisms controlling the subcellular localization of the dUSP36 isoforms, truncated constructs were produced (Figure 2A) and transfected into S2 cells: the 110–1038 construct which corresponds to the common part of the isoforms is localized in the nucleus (Figure 2C), as is the 477-1038 C-terminal construct (Figure 2E). On the opposite, the 110–670 construct, which contains the USP catalytic domain, is not only present in the nucleus but also in the cytoplasm (Figure 2D). These data place the sequence(s) responsible for the nuclear localization of the dUSP36-C and -D isoforms in the 670–1038 C-terminal domain, which is consistent with the identification of two putative Nuclear Localization Sequences (NLS, represented by black bars in Figure 2A) by NLS prediction programs (PSORT, NLS Mapper and SeqNLS). As these sequences are also present in the dUSP36-B isoform that is not localized in the nucleus, we hypothesized that the N-terminal forty amino-acid long domain specific of this isoform (Figure 2B) acts as a Nuclear Export Sequence (NES). Nuclear export of proteins occurs either through the classical nuclear export pathway mediated by the evolutionarily conserved CRM1 protein or through non-classical export pathways mediated by other importin β members. CRM1-dependent NESs are leucine-rich and typically contain large hydrophobic conserved residues separated by a variable number of amino acids (Fasken et al., 2000; Henderson and Eleftheriou, 2000; Xu et al., 2012). The primary sequence of the dUSP36-B specific domain is enriched in leucine residues and contains a CRM1-dependent NES consensus sequence (Figure 2B). This domain was fused to the transcription factor Relish (REL). Compared to the REL protein which is localized in the cell nucleus (Figure 2F), the fusion protein is excluded from the nucleus and accumulates in the cytoplasm (Figure 2G), demonstrating that the N-terminal domain specific of the dUSP36-B isoform has a NES activity.

These data show that the *dUsp36* gene encodes two nuclear isoforms (dUSP36-C and -D) and one cytoplasmic isoform (dUSP36-B) which is exported from the nucleus due to the presence of a NES in its specific N-terminal domain.

### Isoform-Specific Mutations of the *dUsp36* Gene

To specifically inactivate each one of the dUSP36 isoforms, we performed three CRISPR-Cas9 mutagenesis targeting each one of the isoform-specific exons downstream of their respective ATGs (Yu et al., 2013; Port et al., 2014) (Supplementary Figure S1) and retained one nucleotide deletions inducing frameshift mutations for each one of the three isoforms (Figures 1A,E). In all subsequent experiments, each isoform-specific mutation has been analyzed in trans over the null *dUsp36^Δ43^* allele. This allele was previously generated by *P* element excision (Thevenon et al., 2009) and corresponds to a deletion of most of the *dUsp36* gene, thus affecting all three isoforms (Figure 1A).

Western blot analysis using a specific anti-dUSP36 antibody (Buszczak et al., 2009) revealed that the *dUsp36* gene produces the three isoforms at their expected size (Figure 1F). In *dUsp36-B* mutants, the dUSP36-B isoform is missing whereas the other two isoforms are normally expressed while in *dUsp36-C* and *-D* mutants, the dUSP36-C and -D isoforms are specifically absent, respectively (Figure 1F). These results show that each frameshift mutation efficiently and specifically inactivates the expected dUSP36 isoform.

**FIGURE 3:**
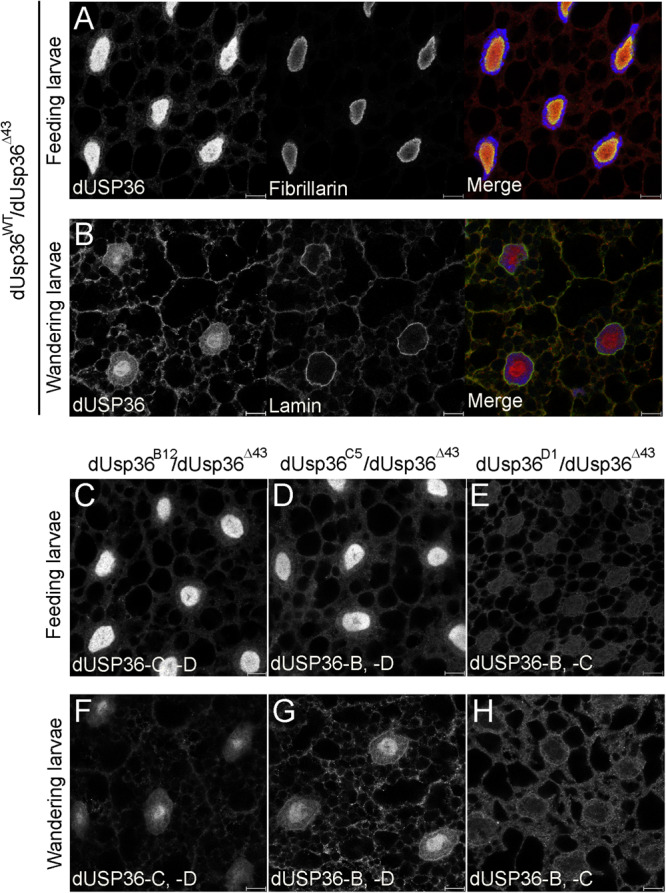
Subcellular localization of the dUSP36 isoforms *in vivo*. Fat bodies of wild-type feeding **(A)** and wandering **(B)** third-instar larvae stained with a specific anti-dUSP36 antibody (Buszczak et al., 2009) and either an anti-Fibrillarin **(A)** or an anti-Lamin **(B)** antibody are shown as separate gray channels and as merged images [Blue: Hoechst. Red: anti-dUSP36 antibody. Green: anti-Fibrillarin **(A)** or anti-Lamin **(B)** antibodies]. **(C–H)** Fat bodies of feeding **(C–E)** and wandering **(F–H)** larvae of the indicated genotype stained with a specific anti-dUSP36 antibody (Buszczak et al., 2009) are shown. For each mutant, the remaining dUSP36 isoforms are indicated for clarity. Scale bar: 10 μm.

These isoform-specific mutations were then used to study the subcellular localization of the dUSP36 isoforms *in vivo*. In fat body cells of wild-type feeding third-instar larvae, the dUSP36 protein, visualized with a specific anti-dUSP36 antibody recognizing the three isoforms (Buszczak et al., 2009), is mainly nucleolar as evidenced by colocalization with the nucleolar marker Fibrillarin (Figure 3A). In *dUsp36-B* and *-C* mutants, no clear modification of dUSP36 expression is observed (Figures 3C,D) whereas in *dUsp36-D* mutants, the nucleolar expression of dUSP36 disappears and a faint residual expression is observed throughout the cell which may correspond to dUSP36-B or -C expression (Figure 3E).

Later in development, in wandering third-instar larvae, dUSP36 expression decreases in the nucleolus and increases both in the cytoplasm and at the nuclear membrane, as shown by colocalization with Lamin (Figure 3B). In *dUsp36-B* mutants, the cytoplasmic and perinuclear expression of dUSP36 is lost whereas the protein is still present in the nucleolus (Figure 3F). In contrast, in *dUsp36-D* mutants, only the nucleolar expression is affected whereas the cytoplasmic and perinuclear accumulation of dUSP36 is still observed (Figure 3H). Finally, as observed in younger larval fat body cells, the mutation of the dUSP36-C isoform does not significantly affect dUSP36 expression (Figure 3G).

Altogether, these data show that the dUSP36-B isoform is present in the cytoplasm and at the nuclear membrane whereas the dUSP36-D isoform is nucleolar, as observed for the human USP36 protein (Endo et al., 2009; Sun et al., 2015a). The dUSP36-C isoform is either not expressed in the larval fat body cells or at very low level that could contribute to the residual nuclear staining observed in *dUsp36-D* mutant cells (Figures 3E,H).

### The Nucleolar dUSP36-D Isoform Is Required for Cell and Organismal Growth *in vivo*

**FIGURE 4:**
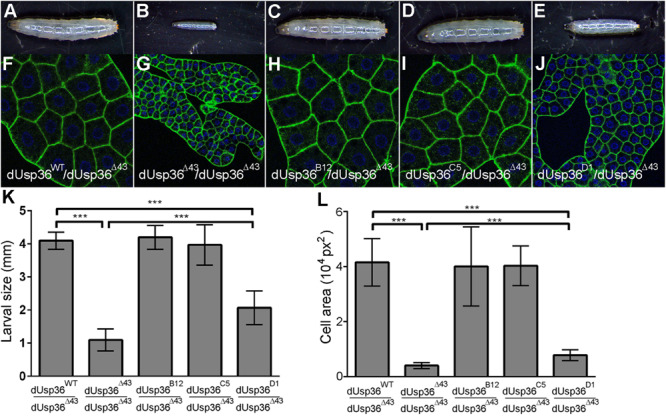
Larval phenotypes of *dUsp36* isoform-specific mutants. **(A–E)** Four day-old *Drosophila* larvae and **(F–J)** confocal sections of dissected fat bodies stained with DAPI (blue) and phalloidin (green) of the indicated genotype. Quantification and statistical analysis of the larval size **(K)** and fat body cell area **(L)** of *dUsp36* isoform-specific mutants. *** Indicates that the statistical analysis by *T* test produces a *P*-value lower than 0.001.

Analysis of the developmental effects of the isoform-specific mutations revealed that inactivation of the dUSP36-B and -C isoforms does not affect larval size (Figures 4C,D,K) whereas the specific mutation of the dUSP36-D isoform results in smaller larvae (Figures 4E,K). This growth phenotype is correlated with a strong reduction of the size of the larval fat body cells (Figures 4J,L). However, this growth defect is milder than the one observed in *dUsp36* null mutants (Figures 4B,G,K,L).

**FIGURE 5:**
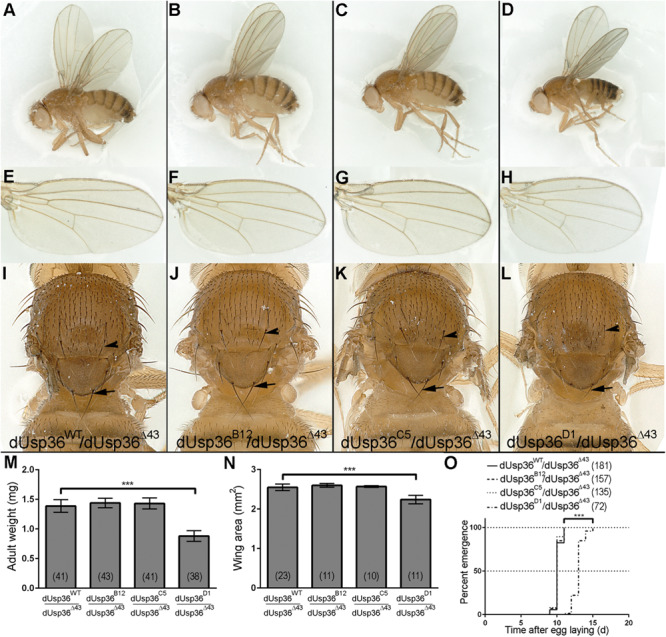
Adult phenotypes of *dUsp36* isoform-specific mutants. **(A–D)** Adult *Drosophila* females, **(E–H)** wings, and **(I–L)** thorax close-ups of the indicated genotype. The arrows and arrowheads point respectively at scutellar and dorsocentral bristles. Quantifications and statistical analyses of the adult weight **(M)**, wing area **(N)** and developmental delay **(O)** of *dUsp36* isoform-specific mutants. The bracketed numbers indicate the number of flies **(M,O)** and wings **(N)** analyzed for each genotype. *** Indicates that the statistical analysis by *T*-test **(M,N)** or Log-rank test **(O)** produces a *P*-value lower than 0.001.

While *dUsp36* null mutants die during larval stages (Taillebourg et al., 2012), isoform-specific mutants are all viable (Figure 5). *dUsp36-B* and *-C* mutants do not display any growth defects or developmental delay (Figures 5B,C,M–O). This is not the case of the *dUsp36-D* mutants, which are smaller than control flies (Figures 5D,M) and have smaller wings (Figures 5H,N). In addition, they have shorter and thinner scutellar (arrows) and dorsocentral (arrowheads) bristles (Figure 5L) and display delayed development (Figure 5O).

Altogether, these results show that the dUSP36-D isoform is the main isoform involved in cell and organismal growth. The weaker phenotype of *dUsp36-D* mutants compared to null *dUsp36* mutants suggests that the residual expression of dUSP36-B and/or -C isoforms may marginally contribute to cell growth, at least in the absence of dUSP36-D.

### dUSP36-D Interacts With dMYC and the E3 Ligase AGO

**FIGURE 6:**
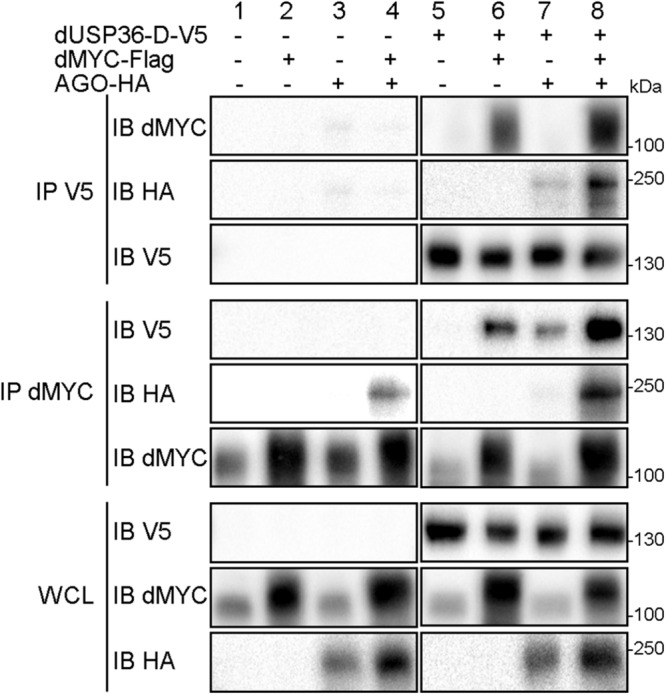
dUSP36-D interacts with dMYC and AGO. *Drosophila* S2 cells were transfected with mock, dUSP36-D-V5, dMYC-Flag and/or AGO-HA plasmids. Whole cell lysates (WCL) were analyzed either directly by Western blot or after immunoprecipitation (IP), and immunoblotted (IB) with the indicated antibodies.

We next investigated the molecular interactions between the dUSP36-D isoform, dMYC and the E3 ligase AGO for numerous reasons. First, *dUsp36-D* and *dUsp36* null mutants are phenotypically very similar to hypomorphic and null *dMyc* mutants respectively (Johnston et al., 1999; Pierce et al., 2004; Gallant, 2013; Grifoni and Bellosta, 2015). Moreover, human USP36 has been shown to regulate c-MYC stability in the nucleolus by antagonizing the activity of the E3 ligase Fbw7γ (Sun et al., 2015a). Lastly, the *Drosophila* Fbw7γ ortholog AGO is known to regulate dMYC stability (Moberg et al., 2004). Co-immunoprecipitation experiments with dUSP36-D show that it interacts with dMYC (Figure 6, panel IP V5 IB dMYC, lanes 6 and 8) and AGO (Figure 6, panel IP V5 IB HA, lanes 7 and 8). As shown previously (Moberg et al., 2004), we also observe an interaction between dMYC and AGO (Figure 6, panel IP dMYC IB HA, lanes 4 and 8). These data demonstrate that dUSP36-D, dMYC and AGO interact with each other. However, they do not tell whether these interactions take place as three different heterodimers or if these proteins are part of the same complex. Interestingly, AGO overexpression strengthens the interaction between dMYC and dUSP36-D (Figure 6, panel IP dMYC IB V5, compare lane 6 to lane 8). Moreover, immunoprecipitation of the endogenous dMYC protein (Figure 6, panel IP dMYC IB dMYC, lanes 1, 3, 5, and 7) allows the co-precipitation of dUSP36-D only when AGO is overexpressed (Figure 6, panel IP dMYC IB V5, compare lane 7 to lane 5). Taken together these data strongly argue that dUSP36-D, dMYC and AGO are part of the same macromolecular complex.

### dUSP36-D Deubiquitinates and Stabilizes dMYC

**FIGURE 7:**
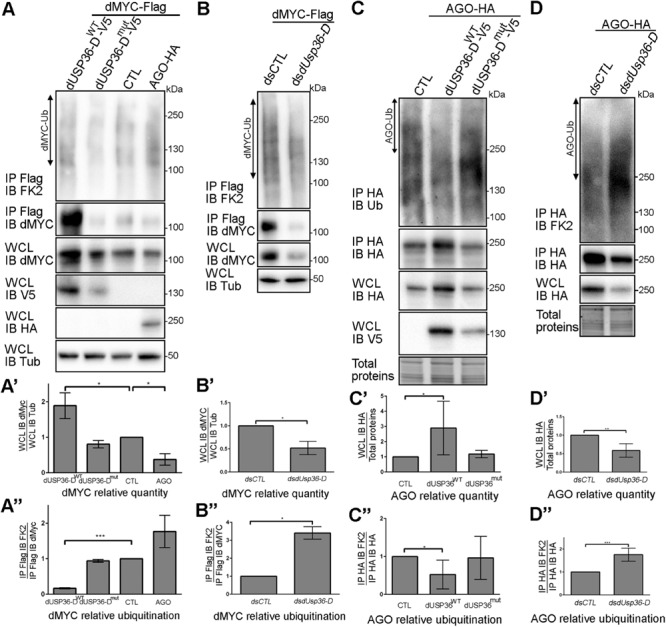
dUSP36-D deubiquitinates and stabilizes dMYC. **(A)** *Drosophila* S2 cells were transfected with a dMYC-Flag expressing plasmid and with either empty (CTL), wild-type dUSP36-D-V5 (dUSP36-D^WT^), catalytic-dead dUSP36-D-V5 (dUSP36-D^mut^) or AGO-HA expressing plasmids. Whole cell lysates (WCL) were analyzed either directly by Western blot or after immunoprecipitation (IP), and immunoblotted (IB) with the indicated antibodies. **(A’)** Quantification of dMYC relative quantity as the ratio of the signal intensity of dMYC over the signal intensity of IB Tub (Tubulin) in WCL. *N* = 3. **(A”)** Quantification of dMYC relative ubiquitination as the ratio of the signal intensity of IB FK2 over the signal intensity of IB dMYC in IP Flag (dMYC). *N* = 3. **(B)** *Drosophila* S2 cells were transfected with a dMYC-Flag expressing plasmid and with either a non-target dsRNA (dsCTL) or a dsRNA targeting the dUSP36-D isoform (*dsdUsp36*-D). **(B’)** Quantification of dMYC relative quantity as the ratio of the signal intensity of dMYC over the signal intensity of IB Tub (Tubulin) in WCL. *N* = 3. **(B”)** Quantification of dMYC relative ubiquitination as the ratio of the signal intensity of IB FK2 over the signal intensity of IB dMYC in IP Flag (dMYC). *N* = 3. **(C)** *Drosophila* S2 cells were transfected with an AGO-HA expressing plasmid and with either empty (CTL), wild-type dUSP36-D-V5 (dUSP36-D^WT^) or catalytic-dead dUSP36-D-V5 (dUSP36-D^mut^) expressing plasmids. **(C’)** Quantification of AGO relative quantity as the ratio of the signal intensity of IB HA (AGO) over the signal intensity of total proteins in WCL. *N* = 5. **(C”)** Quantification of AGO relative ubiquitination as the ratio of the signal intensity of IB FK2 over the signal intensity of IB HA (AGO) in IP HA (AGO). *N* = 6. **(D)** *Drosophila* S2 cells were transfected with an AGO-HA expressing plasmid and with either a non-target dsRNA (*dsCTL*) or a dsRNA targeting the dUSP36-D isoform (*dsdUsp36-D*). **(D’)** Quantification of AGO relative quantity as the ratio of the signal intensity of IB HA (AGO) over the signal intensity of total proteins in WCL. *N* = 4. **(D”)** Quantification of AGO relative ubiquitination as the ratio of the signal intensity of IB FK2 over the signal intensity of IB HA (AGO) in IP HA (AGO). *N* = 6. ***, **, and * indicate that the statistical analysis by *T*-test produces a *P*-value lower than 0.001, 0.01, and 0.05, respectively.

We then asked whether dMYC quantity and ubiquitination levels are regulated by dUSP36-D and AGO. To this end, the dMYC protein was expressed alone (CTL) or in combination with the wild-type dUSP36-D protein (dUSP36-D^WT^), a mutated version of dUSP36-D devoid of catalytic activity (dUSP36-D^mut^) or the E3 ligase AGO (Figure 7A). As already described (Moberg et al., 2004), when AGO is expressed dMYC quantity is decreased (Figures 7A,A’) and its ubiquitination is increased (Figures 7A,A”). On the opposite, when the wild-type dUSP36-D protein is expressed, dMYC quantity is strongly increased (Figures 7A,A’) which is correlated with a sharp drop of its ubiquitination levels (normalized according to the quantity of immunoprecipitated dMYC) (Figures 7A,A”). Expression of the mutated dUSP36-D protein has no significant effect on dMYC quantity nor on its ubiquitination levels (Figures 7A–A”) indicating that dUSP36-D acts on dMYC through its catalytic activity.

Loss of function experiments were also performed using specific dsRNAs targeting the dUSP36-D isoform in cells overexpressing dMYC (Figure 7B). Silencing of *dUsp36-D* significantly decreases the quantity of overexpressed dMYC (Figures 7B,B’) and increases its ubiquitination level (Figures 7B,B”). Taken together, these results show that dUSP36-D deubiquitinates and stabilizes dMYC.

### dUSP36-D Deubiquitinates and Stabilizes the E3 Ligase AGO

The interaction between dUSP36-D and AGO (Figure 6) prompted us to investigate whether AGO is also a substrate of dUSP36-D. To test this hypothesis, a tagged version of AGO was expressed in S2 cells alone (CTL) or in combination with either the wild-type dUSP36-D protein or its mutated version (Figure 7C). The wild-type dUSP36-D protein, but not the mutated form, significantly increases the quantity of AGO protein (Figures 7C,C’) and decreases its ubiquitination (Figures 7C,C”), suggesting that AGO is indeed a substrate of dUSP36-D catalytic activity. Loss of function experiments strengthen this conclusion since silencing of *dUsp36-D* using an isoform-specific dsRNA diminishes the quantity of AGO protein (Figures 7D,D’) and increases its ubiquitination (Figures 7D,D”). These data thus show that dUSP36-D deubiquitinates and stabilizes AGO.

### dUSP36-D Is Ubiquitinated by the E3 Ligase AGO and Deubiquitinated by Itself

**FIGURE 8:**
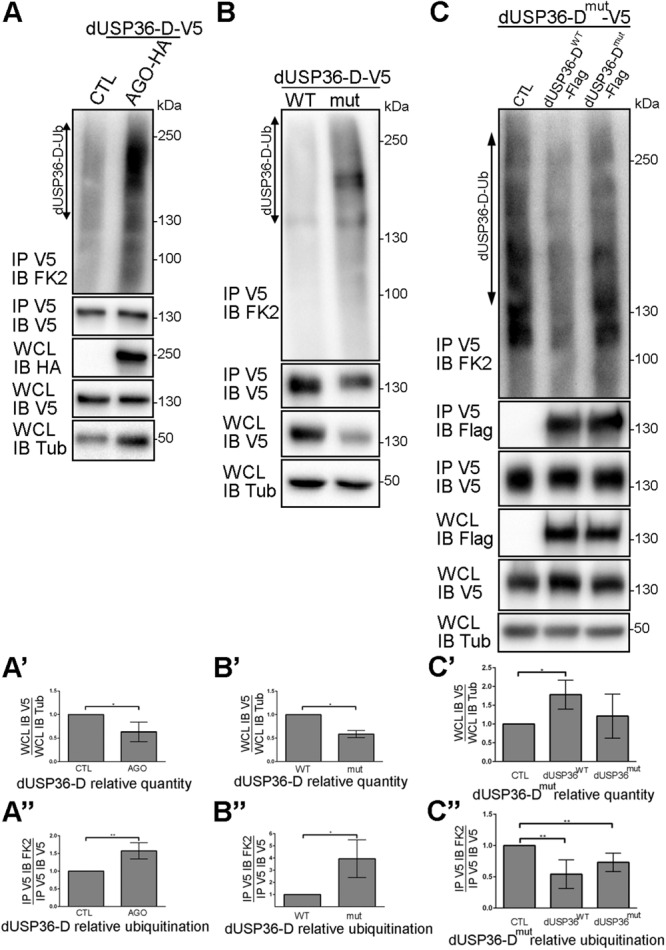
dUSP36-D is ubiquitinated by the E3 ligase AGO and deubiquitinated by itself. **(A)** *Drosophila* S2 cells were transfected with a dUSP36-D-V5 expressing plasmid and with either empty (CTL) or AGO-HA expressing plasmids. Whole cell lysates (WCL) were analyzed either directly by Western blot or after immunoprecipitation (IP), and immunoblotted (IB) with the indicated antibodies. **(A’)** Quantification of dUSP36-D relative quantity as the ratio of the signal intensity of IB V5 (dUSP36-D) over the signal intensity of IB Tub (Tubulin) in WCL. *N* = 3. (**A”)** Quantification of dUSP36-D relative ubiquitination as the ratio of the signal intensity of IB FK2 over the signal intensity of IB V5 (dUSP36-D) in IP V5 (dUSP36-D). *N* = 3. **(B)** *Drosophila* S2 cells were transfected with either wild-type dUSP36-D-V5 (dUSP36-D^WT^) or catalytic-dead dUSP36-D-V5 (dUSP36-D^mut^) expressing plasmids. **(B’)** Quantification of dUSP36-D relative quantity as the ratio of the signal intensity of IB V5 (dUSP36-D) over the signal intensity of IB Tub (Tubulin) in WCL. *N* = 3. **(B”)** Quantification of dUSP36-D relative ubiquitination as the ratio of the signal intensity of IB FK2 over the signal intensity of IB V5 (dUSP36-D) in IP V5 (dUSP36-D). *N* = 3. **(C)** *Drosophila* S2 cells were transfected with a catalytic-dead dUSP36-D-V5 (dUSP36-D^mut^) expressing plasmid and with either empty (CTL), wild-type dUSP36-D-Flag (dUSP36-D^WT^) or catalytic-dead dUSP36-D-Flag (dUSP36-D^mut^) expressing plasmids. **(C’)** Quantification of dUSP36-D^mut^ relative quantity as the ratio of the signal intensity of IB V5 (dUSP36-D^mut^) over the signal intensity of IB Tub (Tubulin) in WCL. *N* = 3. **(C”)** Quantification of dUSP36-D^mut^ relative ubiquitination as the ratio of the signal intensity of IB FK2 over the signal intensity of IB V5 (dUSP36-D^mut^) in IP V5 (dUSP36-D^mut^). *N* = 5. ***, **, and * indicate that the statistical analysis by *T*-test produces a *P*-value lower than 0.001, 0.01, and 0.05, respectively.

S2 cells overexpressing a V5-tagged version of dUSP36-D and treated with the proteasome inhibitor MG132 show an accumulation of dUSP36-D ubiquitinated species (Supplementary Figure S2) suggesting that dUSP36-D is subjected to ubiquitination prior to proteasomal degradation. As the two proteins interact (Figure 6), we investigated the role of the E3 ligase AGO in dUSP36-D ubiquitination. We observed indeed that dUSP36-D levels are decreased by AGO overexpression (Figures 8A,A’) whereas its ubiquitination is concomitantly increased (Figures 8A,A”) indicating that dUSP36-D is ubiquitinated by AGO, which triggers its degradation.

During the course of our experiments, we noticed that the catalytic inactive form of dUSP36-D is systematically present in fewer quantities than the wild-type protein in cell lysates (Figures 7A,C). Quantification of the amount and ubiquitination level of wild-type and mutant dUSP36 proteins confirmed that the catalytically inactive form of dUSP36-D is both less abundant (Figures 8B,B’) and more ubiquitinated than the wild-type protein (Figures 8B,B”). These results suggest that the wild-type dUSP36-D protein is able to regulate its own ubiquitination level.

To test this hypothesis, the V5-tagged catalytic mutant form of dUSP36-D was expressed alone (CTL) or in combination with Flag-tagged wild-type or mutant dUSP36-D proteins (Figure 8C). We observed that the V5-tagged dUSP36-D protein immunoprecipitates the Flag-tagged dUSP36-D proteins (Figure 8C, panel IP V5 IB Flag) indicating that dUSP36-D can dimerize or that multiple copies of dUSP36-D are part of the same complex. Moreover, expression of dUSP36-D^WT^-Flag increases the quantity of dUSP36-D^mut^-V5 (Figures 8C,C’) whereas it decreases its ubiquitination level (Figures 8C,C”) arguing that dUSP36-D is able to deubiquitinate itself.

Taken together, our results show that dUSP36-D is ubiquitinated by the E3 ligase AGO and is able to promote its own deubiquitination (auto-deubiquitination).

### *dUsp36* Genetically Interacts With *dMyc* and *Ago*

Our previous data indicate that dUSP36-D, AGO and dMYC are part of the same complex in which AGO ubiquitinates and destabilizes dMYC and dUSP36-D, and dUSP36-D deubiquitinates and stabilizes dMYC, AGO and itself. dUSP36-D and AGO are thus expected to have antagonistic functions on dMYC-induced cell growth. The *in vivo* relevance of these results was investigated by looking at genetic interactions between *dUsp36*, *dMyc*, and *Ago*.

**FIGURE 9:**
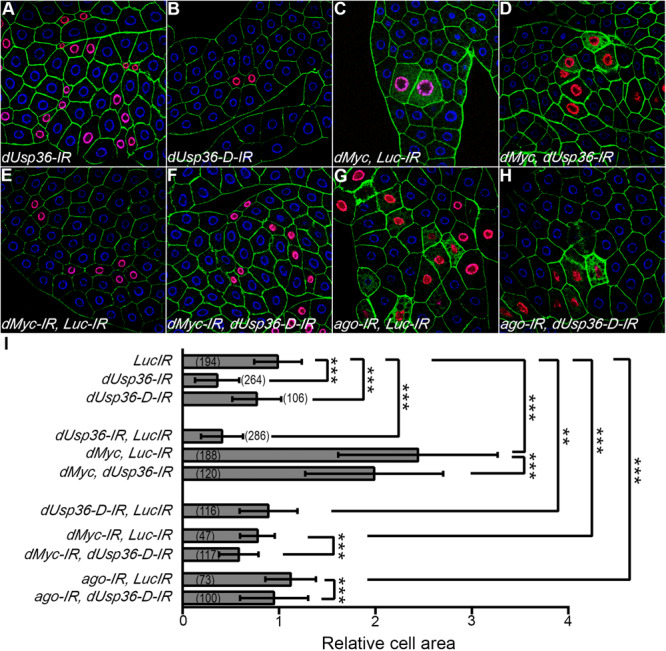
*dUsp36* genetically interacts with *dMyc* and *Ago* in larval fat body cells. **(A–H)** Confocal sections of dissected third instar larvae fat bodies in which the Flpout system was used to clonally express the indicated transgenes along with an HB-RFP transgene. Blue: DAPI. Red: H2B-RFP. Green: Phalloidin **(I)** the ratio between the size of transgene-expressing cells (identified by their red nuclei) and neighboring wild-type cells was calculated for each genotype. The bracketed numbers indicate the number of ratios analyzed. ***, **, and * indicate that the statistical analysis by *T*-test produces a *P*-value lower than 0.001, 0.01, and 0.05, respectively.

To this end, various transgenes were expressed in a few cells of the larval fat body using the Flpout system (Pignoni and Zipursky, 1997) to generate a chimeric tissue and compare the size of the transgene expressing cells to that of wild-type cells in the same context (Figure 9). The transgene expressing cells were easily detected because of the co-expression of an H2B-RFP construct (Langevin et al., 2005). When a control dsRNA transgene targeting the Luciferase gene (*Luc-IR*) is expressed, the ratio between the sizes of the transgene-expressing (red) and the wild-type cells is equal to one (Figure 9I), indicating that cell growth is not affected. This *Luc-IR* transgene was then used to balance the number of transgenes in subsequent experiments. Expressing dsRNA transgenes targeting either all dUSP36 isoforms (Figure 9A) or only the dUSP36-D isoform (Figure 9B) significantly reduces cell size (Figure 9I). As observed in previous experiments (Figure 4), inactivation of all dUSP36 isoforms displays the strongest effect (Figure 9I). In contrast, as previously shown (Parisi et al., 2013), cells overexpressing the dMYC protein show a strong increase in size (Figures 9C,I). Inactivating *dUsp36* in this context significantly suppresses the effect of *dMyc* overexpression (Figures 9D,I). When *dMyc* is silenced using a partially efficient dsRNA transgene, cell size is moderately but significantly reduced (Figures 9E,I). Co-inactivating the dUSP36-D isoform enhances this effect (Figures 9F,I). Finally, inactivating *ago* significantly increases cell size (Figures 9G,I) and co-inactivation of the dUSP36-D isoform counteracts this effect (Figures 9H,I).

**FIGURE 10:**
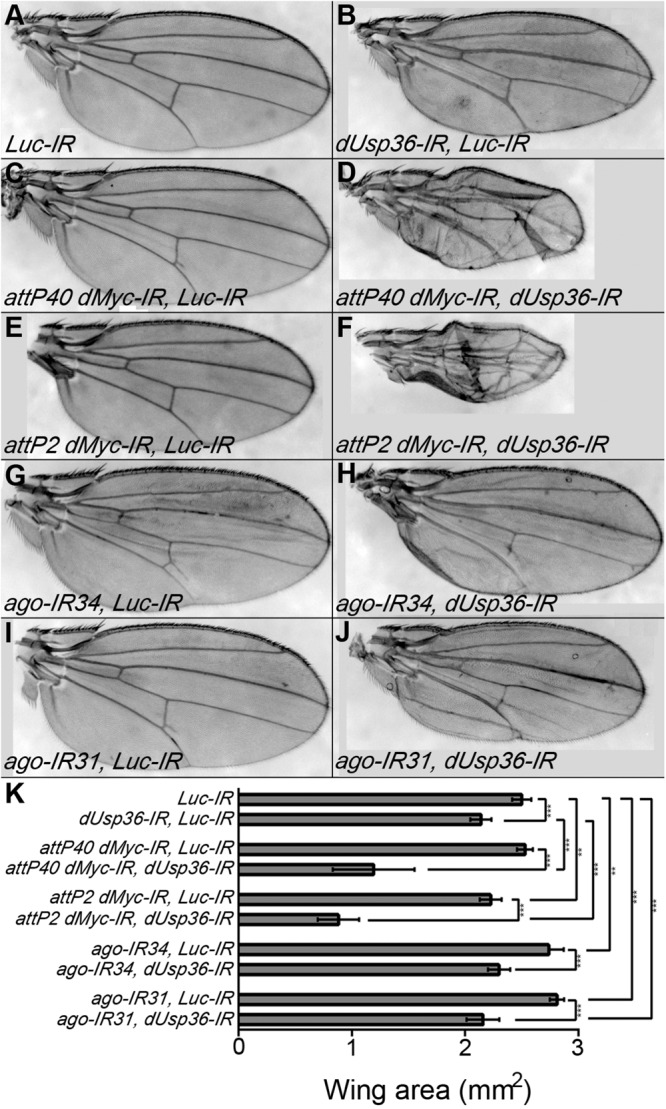
*dUsp36* genetically interacts with *dMyc* and *Ago* in adult wings. **(A–J)** Wings of *Drosophila* females expressing the indicated transgene under the control of the MS1096 wing-specific GAL4 driver. **(K)** Quantification and statistical analysis of the wing area for each genotype. Ten to fourteen wings were measured. ***, **, and * indicates that the statistical analysis by *T*-test produces a *P*-value lower than 0.001, 0.01, and 0.05, respectively.

A second set of genetic interactions was analyzed at the tissue level (Figure 10) using the MS1096 wing-specific GAL4 driver (Brand and Perrimon, 1993; Capdevila and Guerrero, 1994). We observed that silencing of *dUsp36* expression reduces wing size (Figures 10B,K). Two different dsRNA transgenes targeting *dMyc* were also tested for their ability to affect wing size: the first transgene (*attP40 dMyc-IR*, Figure 10C) has no visible effect (Figure 10K) whereas the second (*attP2 dMyc-IR*, Figure 10E) has a moderate but significant effect on wing area (Figure 10K). Although producing no phenotype when expressed alone, the *attP40 dMyc-IR* transgene drastically enhances the wing phenotype induced by the *dUsp36* inactivating transgene (Figures 10D,K), indicating a synergistic relationship between *dMyc* and *dUsp36*. A similar phenotypic enhancement is observed with the *attP2 dMyc-IR* transgene (Figures 10F,K). Lastly, two dsRNA transgenes targeting *ago* were expressed in the wing imaginal disk and result in significantly increased wing size (Figures 10G,I,K). Co-silencing *dUsp36* and *ago* rescues this wing phenotype (Figures 10H,J,K).

These *in vivo* results strongly support the notion that *dMyc* and *dUsp36* act in the same pathway controlling cell growth whereas *dUsp36* and *ago* have antagonistic functions. They are entirely consistent with our previous biochemical experiments. Altogether, our data show that dUSP36-D stabilizes dMYC and promotes cell growth while AGO destabilizes dMYC and inhibits cell growth.

## Discussion

The *dUsp36* gene generates three isoforms differing in their N-terminal domains: we show that when expressed in S2 cells, the C and D isoforms are nuclear whereas the B isoform is localized in the cytoplasm and at the nuclear membrane due to the presence of a NES in its specific N-terminal domain. Isoform-specific mutations generated by CRISPR/Cas9 mutagenesis were then used to infer the subcellular localization of the endogenously expressed isoforms. The dUSP36-B isoform was detected in the cytoplasm and at the nuclear membrane as observed in S2 cells. In contrast, the endogenous dUSP36-D isoform was found to be restricted to the nucleolus whereas it is observed in the whole nucleoplasm when overexpressed in S2 cells or in transgenic *Drosophila* larvae (data not shown). This discrepancy is probably due to the mechanisms of dUSP36-D nucleolar localization being overwhelmed when the protein is overexpressed, pointing out the potential artifacts of overexpression experiments. Our observations are fully consistent with the substrate and function specificity of each isoform: the dUSP36-B isoform, which negatively regulates immune NF-kB-dependent signaling by deubiquitinating the IMD protein (Thevenon et al., 2009) is cytoplasmic whereas the dUSP36-D isoform, which regulates the stability of the dMYC transcription factor is nucleolar. These results illustrate how controlling the subcellular localization of a given DUB gives access to different substrates involved in unrelated functions. These results reinforce the notion that producing DUB isoforms with specific subcellular localizations can greatly expand their functions (Clague et al., 2012, 2013; Leznicki and Kulathu, 2017; Leznicki et al., 2018).

Analysis of the phenotypes induced by the isoform-specific mutations identifies the dUSP36-D isoform as the major contributor of the growth defect of *dUsp36* null mutants. As *dUsp36* and *dMyc* mutant phenotypes are very similar and because human USP36 has been shown to regulate c-MYC stability in the nucleolus, we further characterized the interactions between dUSP36-D, dMYC and its E3 ligase AGO at the biochemical level. We showed (i) that these three proteins are part of the same macromolecular complex, (ii) that AGO ubiquitinates both dMYC, confirming previous results (Moberg et al., 2004), and dUSP36-D and (iii) that dUSP36-D deubiquitinates dMYC, AGO and itself. These results call for several comments. First, we would like to pinpoint the fact that our experiments have been performed by detecting endogenous ubiquitin using a monoclonal anti-ubiquitin antibody rather than overexpressed tagged ubiquitin. Although this approach resulted in weaker ubiquitin signals and did not allow us to perform stringent immunoprecipitations to ascertain that the entire ubiquitin signal is actually due to the immunoprecipitated protein, it ensures to observe physiological levels of ubiquitination. Moreover, these experiments were performed without addition of the proteasomal inhibitor MG132 which allowed us to show that the differences in ubiquitination levels were always correlated with protein stability, comforting our observations. Second, we have shown that ubiquitinated AGO is deubiquitinated by dUSP36-D raising the question of the identity of the E3 ligase that ubiquitinates AGO. It is likely that, as shown for AGO homologs Fbw7 in mammals (Min et al., 2012) and Cdc4 in yeast (Zhou and Howley, 1998; Galan and Peter, 1999; Pashkova et al., 2010), AGO promotes its own ubiquitination in *Drosophila* as well. Finally, we have also shown that dUSP36-D is capable of self-deubiquitination, an ability described for other DUBs (Wada and Kamitani, 2006; Huang et al., 2011; Mashtalir et al., 2014). For example, USP4 associates with the E3 ligase Ro52 forming, as observed here for dUSP36-D and AGO, a DUB/E3 ligase pair that transregulates each other by ubiquitination and deubiquitination (Wada and Kamitani, 2006). DUBs are often found in complexes with E3 ligases (Sowa et al., 2009) and our results strengthen an emerging theme suggesting that these interactions allow DUBs to be included in specific ubiquitin regulating complexes (Leznicki and Kulathu, 2017).

In human, two DUBs have been shown to regulate MYC stability: USP28 in the nucleoplasm (Popov et al., 2007) and USP36 in the nucleolus (Sun et al., 2015a, b). Our results show that, in *Drosophila*, the nucleolar dUSP36-D isoform is required for cell and organismal growth and regulates dMYC stability. However, as no USP28 homolog has been identified in the *Drosophila* genome, the identity of the putative DUB regulating dMYC in the nucleoplasm is unclear. As previously mentioned, we have observed a residual dUSP36 nuclear staining in the *dUsp36-D* mutant cells that could be due to a low expression of the dUSP36-C isoform. However, the lack of growth phenotype of the *dUsp36-C* mutant cells argues against a major role of the dUSP36-C isoform in dMYC stabilization. Alternatively, PUF (puffyeye), which is orthologous to human USP34, is a nuclear DUB that interacts with AGO and regulates dMYC stability in *Drosophila* (Li et al., 2013). It is thus likely that PUF is the DUB regulating dMYC ubiquitination levels and stability in the nucleoplasm. However, *puf* mutants die throughout larval and pupal development with no evidence of growth defects (Li et al., 2013) whereas *dUsp36* null and hypomorphic mutants phenocopy *dMyc* mutants. These results suggest that dUSP36 is the major positive regulator of MYC-dependent cell growth in *Drosophila*. Determining whether dUSP36 and PUF have specialized or redundant functions regarding dMYC regulation will be of interest in the future.

As mentioned above, our data and the fact that AGO homologs display self-ubiquitination activity in mammalian cells (Zhou and Howley, 1998; Galan and Peter, 1999; Pashkova et al., 2010; Min et al., 2012) lead us to propose that dUSP36-D, AGO and dMYC are part of the same complex in which AGO ubiquitinates dMYC, dUSP36-D and itself, and dUSP36-D deubiquitinates dMYC, AGO and itself. Functional interactions in fat body cells and in the wing fully support this model *in vivo*. Moreover, it has been shown that expression of the human USP36 gene is positively regulated by MYC (Sun et al., 2015a) indicating that USP36 and MYC are part of a positive feedback regulatory loop. Interestingly, chromatin immunoprecipitation followed by deep sequencing in *Drosophila* cells showed that dMYC binds to *dUsp36* regulatory regions during interphase but not during mitosis (Yang et al., 2013), indicating a *bona fide* dMYC target gene (Ji et al., 2011). These data suggest that the USP36/MYC positive feedback regulatory loop identified in human is conserved in *Drosophila*.

The results reported here show that the nucleolar dUSP36-D isoform is a major regulator of *dMyc*-dependent cell growth. We also describe a tripartite dMYC-AGO-dUSP36-D complex, which controls dMYC ubiquitination levels and stability in *Drosophila*. This mechanism of regulation is likely conserved in humans, which opens new avenues for a better understanding of its oncogenic deregulation in human cancers.

## Materials and Methods

### Plasmids

The cDNAs for the dUSP36-B (LD40339), -C (AT24152) and -D (AT31021) isoforms were obtained from the *Drosophila* Genomics Resource Center and cloned by PCR into the pAc5.1-V5His plasmid (Invitrogen) to express C-terminally V5-tagged proteins. The dUSP36-D catalytic dead mutant was produced by mutating C181 to S and H439 to N using the QuickChange XL Site-Directed Mutagenesis Kit (Stratagene). The plasmids expressing the tagged AGO (FMO08124) and dMYC (FMO12803) proteins have also been obtained from the *Drosophila* Genomics Resource Center.

### *Drosophila* Experiments

Flies were reared at 25°C except otherwise stated on standard cornmeal–yeast medium.

The *dUsp36-D-IR* plasmid was generated by cloning a hairpin sequence corresponding to the specific 5′ exon of this isoform (sequence available on request) into the pWIZ transgenesis vector (Lee and Carthew, 2003) and transgenic *Drosophila* strains were established (BestGene Inc).

*dUsp36^Δ^^43^*, a null *dUsp36* allele, and the *dUsp36-IR* transgenic line have already been described (Thevenon et al., 2009). The *UAS-Luc-IR* (BL#35788), *UAS-dMyc* (BL#9674), *UAS-dMyc-IR* (BL#43962) and *UAS-ago-IR34* (BL#34802) strains (Ni et al., 2009, 2011) were obtained from the Bloomington *Drosophila* Stock Center. The UAS-H2B-RFP transgenic line (Langevin et al., 2005) was obtained from Dr. Y. Bellaiche.

For the Flpout method, a FRT-flanked cassette blocking expression of the GAL4 gene is excised upon heat-shock induced expression of the FLP recombinase (Pignoni and Zipursky, 1997). This mitotic recombination event leads to the expression of the GAL4 gene and is transmitted across mitosis, generating clones of cells in which GAL4 expression is activated. Spontaneous activation of the GAL4 transcription factor has been reported and allows for the induction of GAL4 expressing cells without heat shock (Hennig et al., 2006).

For CRISPR-Cas9 mutagenesis, the gRNA sequences designed to target each one of the dUSP36 isoforms were selected using the “CRISPR Optimal Target Finder” website^1^. These sequences were cloned into the pCFD3 plasmid (Port et al., 2014). The pCDF3-B, -C or -D plasmids were injected into the *nos-Cas9 CFD2* (Port et al., 2014) recipient strain (BestGene Inc.) and the resulting male founders were crossed with *yw, TM3/TM6* females. Their progenies were then screened either by T7 endonuclease I assay (for B and C) or by phenotype (for D). The presence of the mutations was then confirmed by sequencing. For *dUsp36-B*, the progeny of 30 founder males was screened, six mutations were identified. For *dUsp36-C*, the progeny of 25 founder males was screened, 11 mutations were identified. For *dUsp36-D*, the progeny of 51 founder males was screened, 27 mutations were identified. The sequences of the recovered mutations are given in Supplementary Figure S2. The sequences of the oligonucleotides used for screening and sequencing are available on request.

### Cell Culture, Transfections and Gene Inactivation

*Drosophila* S2 cells were maintained at 25°C in Schneider’s *Drosophila* medium supplemented with 10% heat-inactivated serum (FCS, Invitrogen). DNA transfections were performed 48 h prior to cell lysis using Transfectin (Bio-Rad) according to the manufacturer’s instructions. Gene inactivation was achieved by incubating cells with double strand RNA (dsRNA) for 48 h. DNA templates for dsRNA synthesis were generated by PCR (MEGAscript RNAi kit, Ambion) using the primers designed from Heidelberg Fly Array RNAi libraries^2^.

### Immunoprecipitations and Western Blots

Cell lysis was performed in RIPA buffer (50 mM Tris-HCl, 150 mM NaCl, 1 mM EDTA, 1% IGEPAL, 0.5% sodium deoxycholate) supplemented with a protease inhibitor cocktail (Sigma) and with the pan-deubiquitinases inhibitor PR-619 (Sigma-Aldrich). For co-immunoprecipitation assays, lysates were precleared with Protein A or G-Sepharose beads (Sigma-Aldrich) for 4 h at 4°C with rotation. Immunoprecipitations were performed in RIPA buffer. Immune complexes were precipitated with protein A or G-Sepharose beads with the indicated antibody overnight at 4°C, the beads were washed 4 times with RIPA buffer and bound proteins were eluted using Laemmli Sample Buffer (Bio-Rad) supplemented with 10% of β-mercaptoethanol and boiled. Whole cell lysates were diluted with Laemmli Sample Buffer 4x (Bio-Rad) supplemented with 10% of β-mercaptoethanol, boiled at 95°C for 5 min and directly used for immunoblotting.

Protein lysates and eluates were separated on SDS-PAGE gels (TGX Stain Free from Bio-Rad). The total amount of protein was detected directly in the gels using the Image Lab Stain Free Gel protocol. Proteins were transferred to PVDF membranes (Bio-Rad). Membranes were saturated for 1h in TBS/0.1% Tween-20/5% BSA or skimmed milk, incubated with the appropriate primary antibody for 1 to 3 h at RT or overnight at 4°C in TBS/0.1% Tween-20/1% BSA or skimmed milk before three washes in TBS/0.1% Tween-20 (TBST) and then incubated an additional hour at RT with corresponding secondary antibodies coupled to horse radish peroxidase (HRP). After three rinses with TBST, the membranes were revealed with the Luminata Forte Western HRP substrate (Millipore) using the Chemidoc imaging system (Bio-Rad). Quantifications were performed using the Image Lab software (Bio-Rad). The extent of the smear of ubiquitinated proteins taken into account for quantification is indicated by the double arrows on Figures 6, 7.

The anti-dUSP36 antibody, a kind gift from Dr. M. Buszczak, has been already described (Buszczak et al., 2009) and was used at a 1/2500 dilution. The following antibodies were used following the manufacturer’s instructions: monoclonal anti-V5 (Invitrogen), monoclonal anti-Flag clone M2 (Sigma), rat anti-HA High Affinity (Sigma), rabbit anti-dMYC (Santa Cruz Biotechnology, sc28207), monoclonal anti-ubiquitinated conjugates FK2 (Enzo). The secondary antibodies HRP-coupled goat anti-mouse, -rabbit and -rat are from Sigma.

### Immunochemistry, Image Acquisition and Analysis

For immunocytochemistry, S2 cells were fixed for 10 min in 4% paraformaldehyde, rinsed twice in PBS, blocked for 1 h in PBS, 0.1% Triton X-100, 5% normal goat serum and incubated for 1 h with a rabbit anti-V5 antibody (1/500, Invitrogen) and either with monoclonal anti-Lamin Dm0 ADL84.12 (1/200, Developmental Studies Hybridoma Bank) or anti-Fibrillarin 38F3 (1/500, abcam) antibodies. Lateral lobes of third instar larval fat bodies were dissected in PBS, fixed for 30 min in 4% paraformaldehyde and rinsed twice in PBS. The samples were then blocked for 1 h in PBS, 0.1% Triton X-100, 5% normal goat serum and incubated overnight at 4°C with the anti-dUSP36 (1/200) and either with monoclonal anti-Lamin Dm0 ADL84.12 (1/200, Developmental Studies Hybridoma Bank) or anti-Fibrillarin 38F3 (1/200, abcam) antibodies. Secondary antibodies were coupled to Alexa594 or Alexa488 (1/500, Invitrogen).

After mounting in DAPI-containing Vectashield (Vector Laboratories, H-1200), the samples were imaged with a 40x or 63x magnification (oil immersion) using a Leica TCS SP2 confocal microscope and the LCS software. All the pictures shown are representative of the whole tissue and of the observations made from different animals. Cell areas were automatically measured using the cell image analysis software CellProfiler (Carpenter et al., 2006). The analysis pipelines are available on request.

Third instar larvae, wings, thoraces and adults were imaged using a Keyence VHX-5000 numerical microscope. Larval size and wing area were blindly measured manually using the Fiji/ImageJ software (National Institute of Health).

### Statistical Analysis

All statistical analyses were performed in Prism 6 (GraphPad).

## Data Availability Statement

All datasets generated for this study are included in the article/Supplementary Material.

## Author Contributions

DT performed all the biochemical experiments (immunopreci- pation, ubiquitination assay). IS performed the analysis of the subcellular localization of dUSP36 isoforms in S2 cells. CP performed the PCR screening for identifying isoform-specific *dUsp36* mutants. ET and XC-Y performed the CRSPR/Cas9 mutagenesis and the phenotypic characterization of the isoform-specific *dUsp36* mutants. M-OF and ET designed the experiments and wrote the manuscript.

## Conflict of Interest

The authors declare that the research was conducted in the absence of any commercial or financial relationships that could be construed as a potential conflict of interest.

## Footnotes

1 http://tools.flycrispr.molbio.wisc.edu/targetFinder/

2 http://www.genomernai.org/

## Supplementary Material

FIGURE S1 Sequences of the *dUsp36* isoform-specific mutations induced by CRISPR-Cas9 mutagenesis. The wild-type (WT) and mutant sequences corresponding to the dUSP36-B, -C, and -D isoforms are respectively shown. The ATGs used for each isoform are highlighted in bold. The gRNA sequences used for mutagenesis are highlighted in yellow whereas the PAM sequences are highlighted in red. Multiple alleles were recovered for all isoform-specific *dUsp36* mutations and one nucleotide deletions inducing frameshift mutations were retained for further analysis. In addition to those presented in this study, another allele was kept for each isoform and showed the same phenotype (data not shown). For *dUsp36-D*, the bracketed numbers indicate how many times the same mutation has been isolated independently.

FIGURE S2 dUSP36-D is ubiquitinated. *Drosophila* S2 cells were transfected with the dUSP36-D-V5 expressing plasmid and treated with the proteasome inhibitor MG132 (20 μM for 4 h). Whole cell lysates (WCL) were analyzed either directly by Western blot or after immunoprecipitation (IP), and immunoblotted (IB) with the indicated antibodies.
