# Supplementary material for: A Nucleolar Isoform of the Drosophila Ubiquitin Specific Protease dUSP36 Regulates MYC-Dependent Cell Growth
Source: Front Cell Dev Biol. 2020 Jun 19;8:506. doi: 10.3389/fcell.2020.00506 (PMC7316882; doi:10.3389/fcell.2020.00506)
Supplement: FIGURE S1 — Sequences of the dUsp36 isoform-specific mutations induced by CRISPR-Cas9 mutagenesis. The wild-type (WT) and mutant sequences corresponding to the dUSP36-B, -C, and -D isoforms are respectively shown. The ATGs used for each isoform are highlighted in bold. The gRNA sequences used for mutagenesis are highlighted in yellow whereas the PAM sequences are highlighted in red. Multiple alleles were recovered for all isoform-specific dUsp36 mutations and one nucleotide deletions inducing frameshift mutations were retained for further analysis. In addition to those presented in this study, another allele was kept for each isoform and showed the same phenotype (data not shown). For dUsp36-D, the bracketed numbers indicate how many times the same mutation has been isolated independently. [file Image_1.pdf]

dUsp36-B

```
WT     ATGACTGTGATAATGGTGGACGGGTTCGCCCTGTGGCTGCTCTACAAGCTGTTCCTG
B12    ATGACTGTGATAATGGTGGACGGGTTCGC-CTGTGGCTGCTCTACAAGCTGTTCCTG
       ATGACTGTGATAATGGTGGACGGGTTCG--CTGTGGCTGCTCTACAAGCTGTTCCTG
       ATGACTGTGATAATGGTGGACGGGTT-------agagttCTCTACAAGCTGTTCCTG
       ATGACTGTGATAATGGT--------cgtccattggacgGCTCTACAAGCTGTTCCTG
       ATGACTGTGATAATGGTGGACGGGTTCGCgtctacaaGCTGCTCTACAAGCTGTTCCTG
       ATGACTGTGATAATGGTGGACGGccacatgataaTGTGGCTGCTCTACAAGCTGTTCCTG
```

dUsp36-C

```
WT     ATGCTGCACAGTCCGGTGCCTAGGTACAACAAATGTGTACCCTTTCCAACACTGCGA
C5     ATGCTGCACAGTCCGGT-CCTAGGTACAACAAATGTGTACCCTTTCCAACACTGCGA
       ATGCTGCACAGTCCGGTGC---tGTACAACAAATGTGTACCCTTTCCAACACTGCGA
       ATGCTGCACAGTCCGG----acaGTACAACAAATGTGTACCCTTTCCAACACTGCGA
       ATGCTGCACAGTCCGGTGt----GTACAACAAATGTGTACCCTTTCCAACACTGCGA
       ATGCTGCACAGTCCGGT-----GGTACAACAAATGTGTACCCTTTCCAACACTGCGA
       ATGCTGCACAGT------CCTAGGTACAACAAATGTGTACCCTTTCCAACACTGCGA
       ATGCTGCACAGT------CCTAGGTACAACAAATGTGTACCCTTTCCAACACTGCGA
       ATGCTGCACAGT------CCTAGGTACAACAAATGTGTACCCTTTCCAACACTGCGA
       ATGCTGCACAGTCCGGTG--------CAACAAATGTGTACCCTTTCCAACACTGCGA
       ATGCTGCACAGTCCGGTG--------CAACAAATGTGTACCCTTTCCAACACTGCGA
       ATGCTGCACAGTCCtacacacAGGTACAACAAATGTGTACCCTTTCCAACACTGCGA
```

dUsp36-D

```
WT     ATG(36nt)GTCAACGCAGCGCTGCGCGAATCCCTTGGCGGCAACTCCTCCGCCGGC
D1     ATG(36nt)GTCAACGCAGCGCTGCGCGAAT-CCTTGGCGGCAACTCCTCCGCCGGC   (7)
       ATG(36nt)GTCAACGCAGCGCTGCGCcttg-CCTTGGCGGCAACTCCTCCGCCGGC
       ATG(36nt)GTCAACGCAGCGCTGCGCGttg-CCTTGGCGGCAACTCCTCCGCCGGC
       ATG(36nt)GTCAACGCAGCGCTGCGCGAcaa-CTTGGCGGCAACTCCTCCGCCGGC
       ATG(36nt)GTCAACGCAGCGCTGCGCGAATCgca-GGCGGCAACTCCTCCGCCGGC
       ATG(36nt)GTCAACGCAGCGCTGCGCGAAT----TGGCGGCAACTCCTCCGCCGGC
       ATG(36nt)GTCAACGCAGCGCTGC-------CCTTGGCGGCAACTCCTCCGCCGGC
       ATG(36nt)GTCAACGCAGCGCTGCGCGAATC-------GGCAACTCCTCCGCCGGC   (2)
       ATG(36nt)GTCAACGCAGCGCcg--------CCTTGGCGGCAACTCCTCCGCCGGC
       ATG(36nt)GTCAACGCAGCGC----------CCTTGGCGGCAACTCCTCCGCCGGC   (2)
       ATG(36nt)GTCAACGCAGCGC-----------CTTGGCGGCAACTCCTCCGCCGGC
       ATG(36nt)GTCAACGCAGCGCTG-------------GCGGCAACTCCTCCGCCGGC
       ATG(36nt)GTCAACGCAGCGCTGCGCGAATC-------------TCCTCCGCCGGC
       ATG(36nt)GTCAACGCAGCGCTGCGCGAATtgCCTTGGCGGCAACTCCTCCGCCGGC
       ATG(36nt)GTCAACGCAGCGCTGCGCGAgttgCCTTGGCGGCAACTCCTCCGCCGGC
       ATG(36nt)GTCAACGCAGCGCTGCGCGAAgttgCCTTGGCGGCAACTCCTCCGCCGGC
       ATG(36nt)GTCAACGCAGCGCTGCGCGAATtgttgattaCCTTGGCGGCAACTCCTCCGCCGGC
       ATG(36nt)GTCAACGCAGCGCTGCGCGAATgccttgttgCCTTGGCGGCAACTCCTCCGCCGGC
       ATG(36nt)GTCAACGCAGCGCTGCGCGAAccaattacgaaaCCCTTGGCGGCAACTCCTCCGCCGGC
```
