# Supplementary figures and images for: A Nucleolar Isoform of the Drosophila Ubiquitin Specific Protease dUSP36 Regulates MYC-Dependent Cell Growth
Source: Front Cell Dev Biol. 2020 Jun 19;8:506. doi: 10.3389/fcell.2020.00506 (PMC7316882; doi:10.3389/fcell.2020.00506)

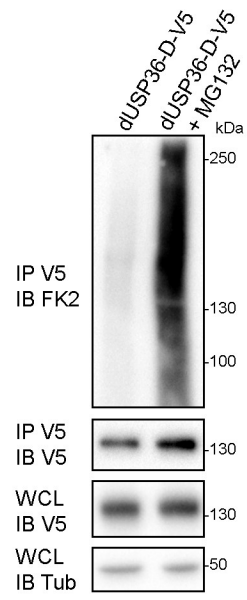

Supplement: FIGURE S2 — dUSP36-D is ubiquitinated. Drosophila S2 cells were transfected with the dUSP36-D-V5 expressing plasmid and treated with the proteasome inhibitor MG132 (20 μM for 4 h). Whole cell lysates (WCL) were analyzed either directly by Western blot or after immunoprecipitation (IP), and immunoblotted (IB) with the indicated antibodies. [file Image_2.pdf]
